# A SNP in pri-miR-10a is associated with recurrent spontaneous abortion in a Han-Chinese population

**DOI:** 10.18632/oncotarget.7002

**Published:** 2016-01-25

**Authors:** Ying Li, Xue-Qin Wang, Lu Zhang, Xiao-Dan Lv, Xing Su, Shi Tian, Chun-Mei Liu, Xu Ma, Hong-Fei Xia

**Affiliations:** ^1^ Reproductive and Genetic Center of National Research Institute for Family Planning, Beijing, China; ^2^ Graduate School, Peking Union Medical College, Beijing, China; ^3^ Haidian Maternal & Child Health Hospital, Beijing, China

**Keywords:** miR-10a, recurrent spontaneous abortion, haplotype, rs3809783

## Abstract

*MicroRNA-10a (miR-10a)* has a wide range of functions in nearly all mammalian tissues and is involved in the occurrence of many diseases. However, it remains unknown whether *miR-10a* is associated with human recurrent spontaneous abortion (RSA). In this study, we found that rs3809783 A > T in *miR-10a* coding region was significantly associated with the increase of the risk of human unexplained RSA (URSA) acquisition in a Han-Chinese population. The T allele of rs3809783 hindered the production of mature miR-10a. A to T substitution in *miR-10a* rs3809783 repressed cell proliferation and migratory capacity. Further investigation discovered that Bcl-2-interacting mediator (*Bim*) was the functional target of *miR-10a* and inversely regulated *Bim* expression. Dual-luciferase assay indicated that A allele in *miR-10a* rs3809783 could more effectively suppress *Bim* expression than T allele. In addition, A to T substitution in *miR-10a* rs3809783 attenuated the sensibility of cells to progesterone and its antagonist mifepristone. Collectively, our data suggest that rs3809783 A > T in pri-miR-10a may be conductive to the genetic predisposition to RSA by disrupting the production of mature *miR-10a* and reinforcing the expression of *Bim*.

## INTRODUCTION

Recurrent spontaneous abortion (RSA) is definded as two or more times consecutive spontaneous abortions before 24 gestational weeks. In the worldwide, about 1–5% of the reproductive age couples are affected by RSA [1]. Genetic factors are a cause of RSA acquisition and include chromosomal abnormalities, gene polymorphisms and so on [2, 3]. The regions of genomes polymorphisms associated with RSA are mainly focused on coding RNAs. Limited knowledge is available about the association between polymorphisms in non-coding RNAs and RSA.

MicroRNAs (miRNAs) are non-coding small RNA molecules that regulate the gene expression by base pairing to partially complementary sites of the target mRNA for degradation or translational repression [4]. It is now acknowledged that miRNAs are associated with many human disease [5]. Recent studies have clearly demonstrated that single nucleotide polymorphisms (SNPs) within the miRNA sequence may affect miRNA processing and modulate miRNA expression [6], eventually lead to the occurrence of diseases [7]. Our previous study has found that *miR-125a* and *miR-10a* are differentially expressed in rat uterus between the implantation and the pre-implantation period [8]. The implantation failure is one of major reasons for recurrent spontaneous abortion. Moreover, two common SNPs (rs41275794, rs12976445) in pri-miR-125a can increase the risk of RSA by decreasing *miR-125a* expression [9]. And it will be an interesting question whether the polymorphisms of *miR-10a* are associated with RSA.

In this study, we explored the possible relationship between the SNP in pri-miR-10a and RSA. We found significant differences in genotype distribution of rs3809783 in pri-miR-10a between RSA patients and normal controls. A to T substitution in *miR-10a* rs3809783 was confirmed to be associated with an increased risk of RSA, which could decrease the production of mature *miR-10a* and inhibit cell growth.

## RESULTS

### Genetic case-control association study

**Figure 1:**
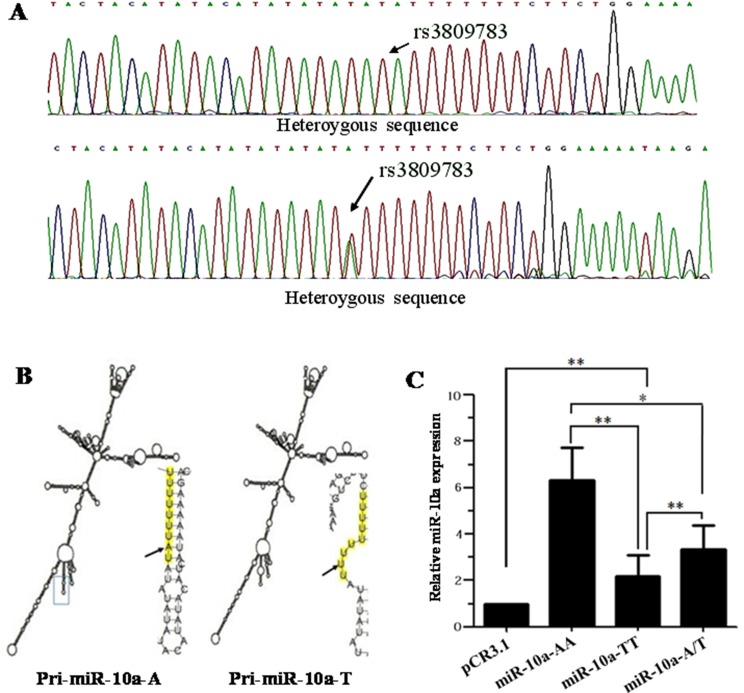
Sequencing, secondary structure prediction and *miR-10a* expression detection (**A**) An example of chromatographs showing the rs3809783 in pri-miR-10a. Black arrows indicate SNP site. (**B**) Predicted structure of pri-miR-10a. RNA fold web server was used to predict the secondary structure of pri-miR-10a. (**C**) *MiR-10a* expression analysis. *MiR-10a* level was detected in cells transfected with the empty pCR3.1 vector, pCR3.1-miR-10a-AA, pCR3.1-miR-10a-TT or pCR3.1-miR-10a-A/T by TaqMan miRNA RT-Real Time PCR. *U6* snRNA was used as internal control. The relative level of *miR-10a* was normalized to U6. **P < 0.01.

According to the data from NCBI dbSNP database, three SNPs (rs72631828, rs3809783, rs34242602) were predicted in the region of pri-miR-10a from −200 bp to +200 bp relative to pre-miR-10a sequence. However, only one SNP (rs3809783) located at position +22 relative to pre-miR-10a was found in the region in Chinese Han women (Table 1 and Figure 1A). The genotype distribution of *miR-10a* rs3809783 in controls was conformed to be in Hardy-Weinberg equilibrium. Compared with controls, the genotype and allele distribution of *miR-10a* rs3809783 in RSA patients were evident differences. A/T heterozygosity of *miR-10a* rs3809783 was significantly associated with an increased risk of RSA (OR = 3.152807, 95% CI = 2.049834∼4.849266, *P* < 0.01).

**Table 1:** The genotype distributions of *miR-10a* rs3809783

	Case (*n* = 200), (n,%)	Control (*n* = 200), (n,%)	OR	OR 95%CI	*P* value
Genotype					
A/A	103 (51.50%)	154 (77.00%)	3.15	2.05–4.85	< 0.01
A/T	97 (48.50%)	46 (23.00%)			
Allele					
A	303(75.75%)	354(88.50%)	2.46	1.68–3.61	< 0.01
T	97 (24.25%)	46 (11.50%)			

OR, odd ratio; 95% CI, 95% confidence interval.

### Secondary structure prediction

RNA fold web server (http://rna.tbi.univie.ac.at/cgi-bin/RNAfold.cgi) was used to predict the secondary structure of 1,020 bp pri-miR-10a sequence including rs3809783 (Figure 1B). The rare T allele changed the loop position and made the loop size bigger than A allele. The predicted thermodynamic ensemble free energy (ΔG) in A allele (−328.20kcal/mol) was higher than that in rare T allele (−302.31 kcal/mol).

### A to T substitution in *miR-10a* rs3809783 hinders the production of mature *miR-10a*

TaqMan miRNA RT-Real Time PCR was used to detect the influence of different genotypes on the production of mature *miR-10a* (Figure 1C). Compared with the A allele, T allele significantly decreased the expression level of mature *miR-10a* (*P* < 0.01). Mature *miR-10a* level was significantly lower in A/T heterozygosity than that in A allele (*P* < 0.05) and higher than that in T allele (*P* < 0.01). These results uncover that A to T substitution in *miR-10a* rs3809783 is not conducive to the production of mature *miR-10a*.

### *MiR-10a* expression and allele in different cell lines

**Figure 2:**
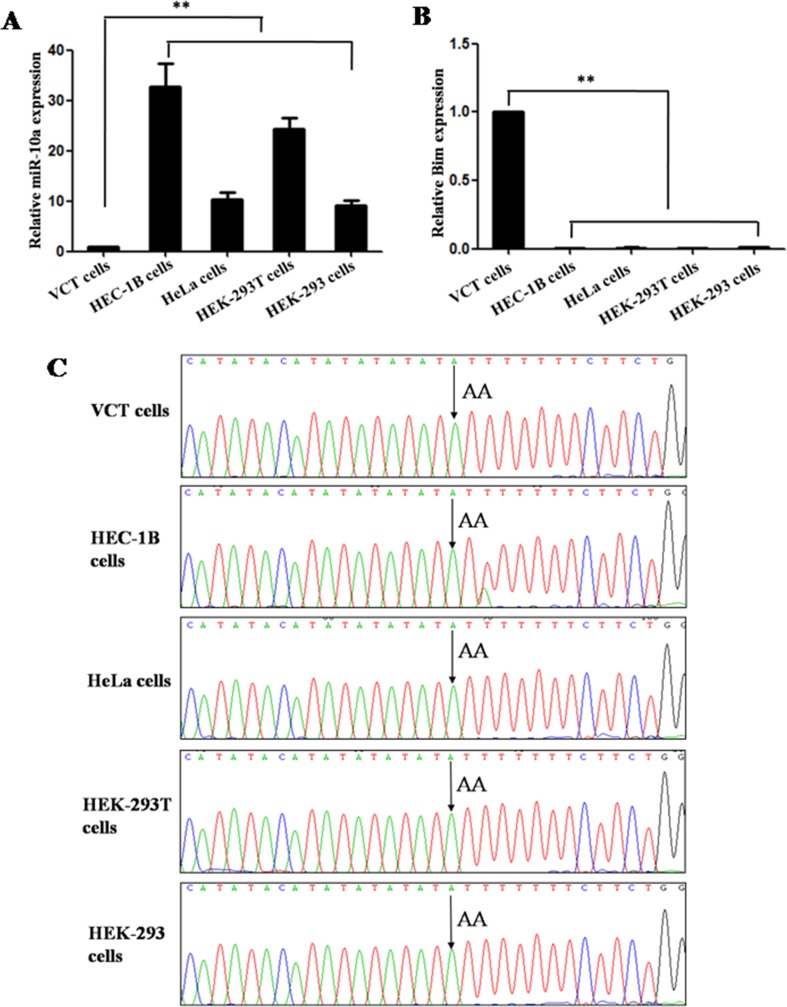
*MiR-10a* expression and allele in different cell lines *MiR-10a* (**A**) and *Bim* expression (**B**) in HEC-1B, HeLa, HEK-293, HEK-293T and VCT cells was detected by qRT-PCR. The allele of *miR-10a* rs3809783 in HEC-1B, HELA, HEK-293, HEK-293T and VCT cells was detected by sequencing (**C**). **P < 0.01.

*MiR-10a* expression in HEC-1B, HeLa, HEK-293, HEK-293T and VCT cells was detected by qRT-PCR. The expression level of *miR-10a* in VCT cells was lower than that in HEC-1B, HeLa, HEK-293 and HEK-293T cells (*P* < 0.01; Figure 2A).

The allele of *miR-10a* rs3809783 in HEC-1B, HELA, HEK-293, HEK-293T and VCT cells was detected by sequencing. It was allele A at position +22 relative to pre-miR-10a sequence (Figure 2C).

### A to T substitution in *miR-10a* rs3809783 inhibits cell proliferation

**Figure 3:**
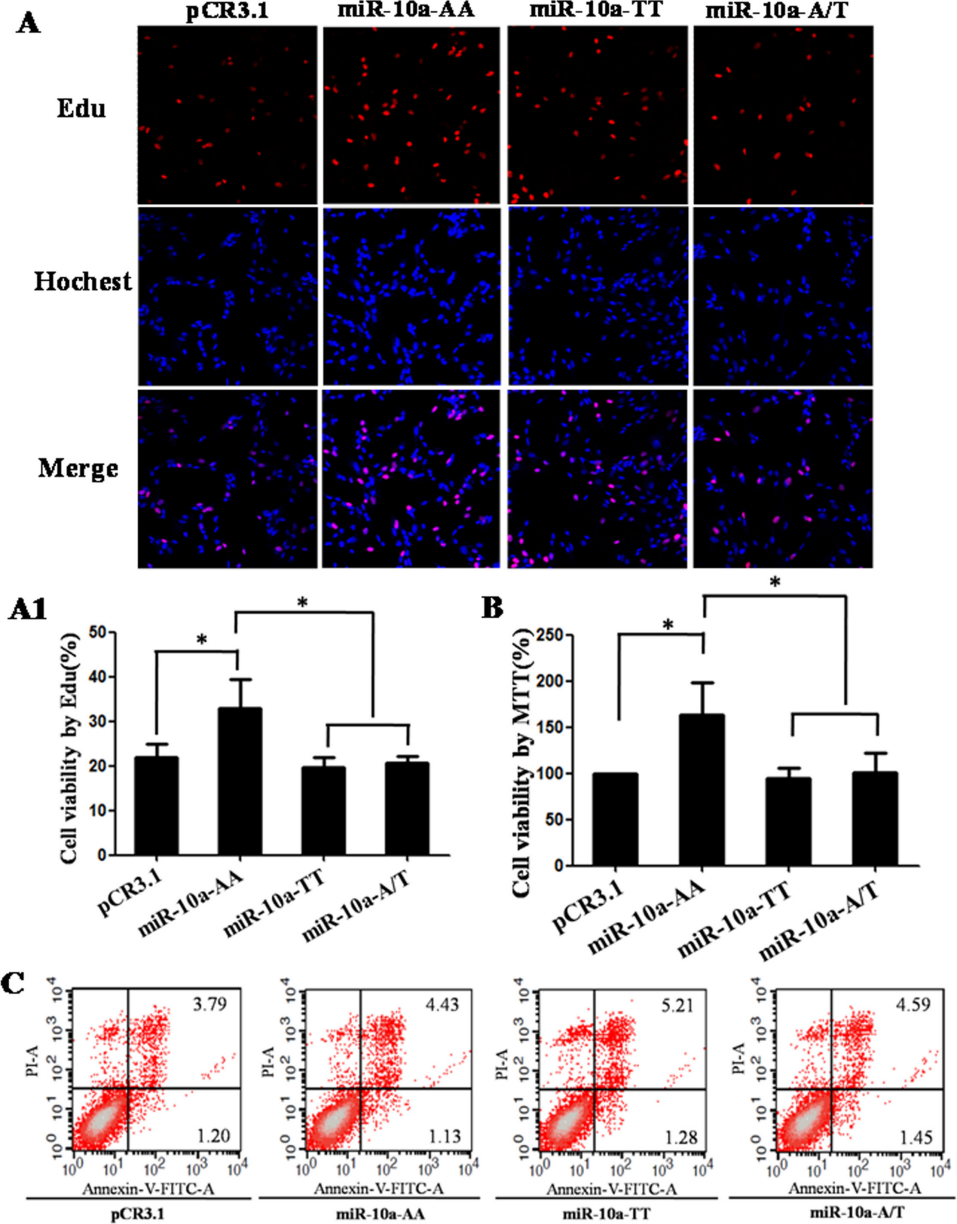
Cell growth analysis in VCT cells transfected by different genotypes pCR3.1, pCR3.1-miR-10a-AA, pCR3.1-miR-10a-TT or pCR3.1-miR-10a-A/T was transfected into VCT cells. After 48 h of transfection, cell proliferation was determined by EdU assay (**A** and **A1**) and MTT assay (**B**). Red represents the proliferative cells. Blue indicates cell nuclei. The photographs were shown at x400 original magnification. Cell apoptosis was detected by flow cytometry analysis (**C**). Lower left quadrant, viable cells (annexin V-FITC and PI negative); lower right quadrant, early apoptotic cells (annexin V-FITC positive and PI negative); upper right quadrant, late apoptosis/necrosis cells (annexin V-FITC and PI positive). The percentage of early and late apoptotic cells was shown in the lower right and upper right panels, respectively. All experiments were performed at least three times.*P < 0.05, **P < 0.01.

The proliferation capacity in VCT cells transfected by different genotypes was detected by Edu assay (Figure 3A and 3A1). Compared with the empty pCR3.1 vector, the AA homozygote significantly enhanced the proliferation rate (*P* < 0.05). The proliferation rates in TT homozygote and A/T heterozygosity were significantly lower than that in AA homozygote (*P* < 0.05). In order to further confirm the role of *miR-10a* rs3809783 in cell viability, the proliferation capacity of VCT cells was determined by MTT assay (Figure 3B). The results were similar with Edu assay. AA homozygote exhibited a significantly higher proliferation rate than the empty pCR3.1 vector (*P* < 0.05), TT homozygote (*P* < 0.05) and A/T heterozygosity (*P* < 0.05). These results show that the rare T allele inhibits cell proliferation relative to A allele.

### A to T substitution in *miR-10a* rs3809783 slightly increases cell apoptosis

The apoptosis in VCT cells transfected with pCR3.1, pCR3.1-miR-10a-AA, pCR3.1-miR-10a-TT and pCR3.1-miR-10a-A/T was determined by flow cytometry analysis (Figure 3C). The percentage of early apoptotic cells (annexin V-FITC positive) in the empty pCR3.1 vector, AA homozygote, TT homozygote and A/T heterozygosity was about 1.20%, 1.13%, 1.28% or 1.45%, respectively. While the percentage of late apoptotic cells (annexin V-FITC/PI positive) in the empty pCR3.1 vector, AA homozygote, TT homozygote and A/T heterozygosity was about 3.79%, 4.43%, 5.21% or 4.59%, respectively. Although the cell apoptotic level in early stage was comparable in different genotypes, the cell apoptotic level in late stage in TT homozygote was slightly higher than that in AA homozygote. These results suggest that the rare T allele has a tendency to promote cell apoptosis in late stage.

### A to T substitution in *miR-10a* rs3809783 represses cell migration

**Figure 4:**
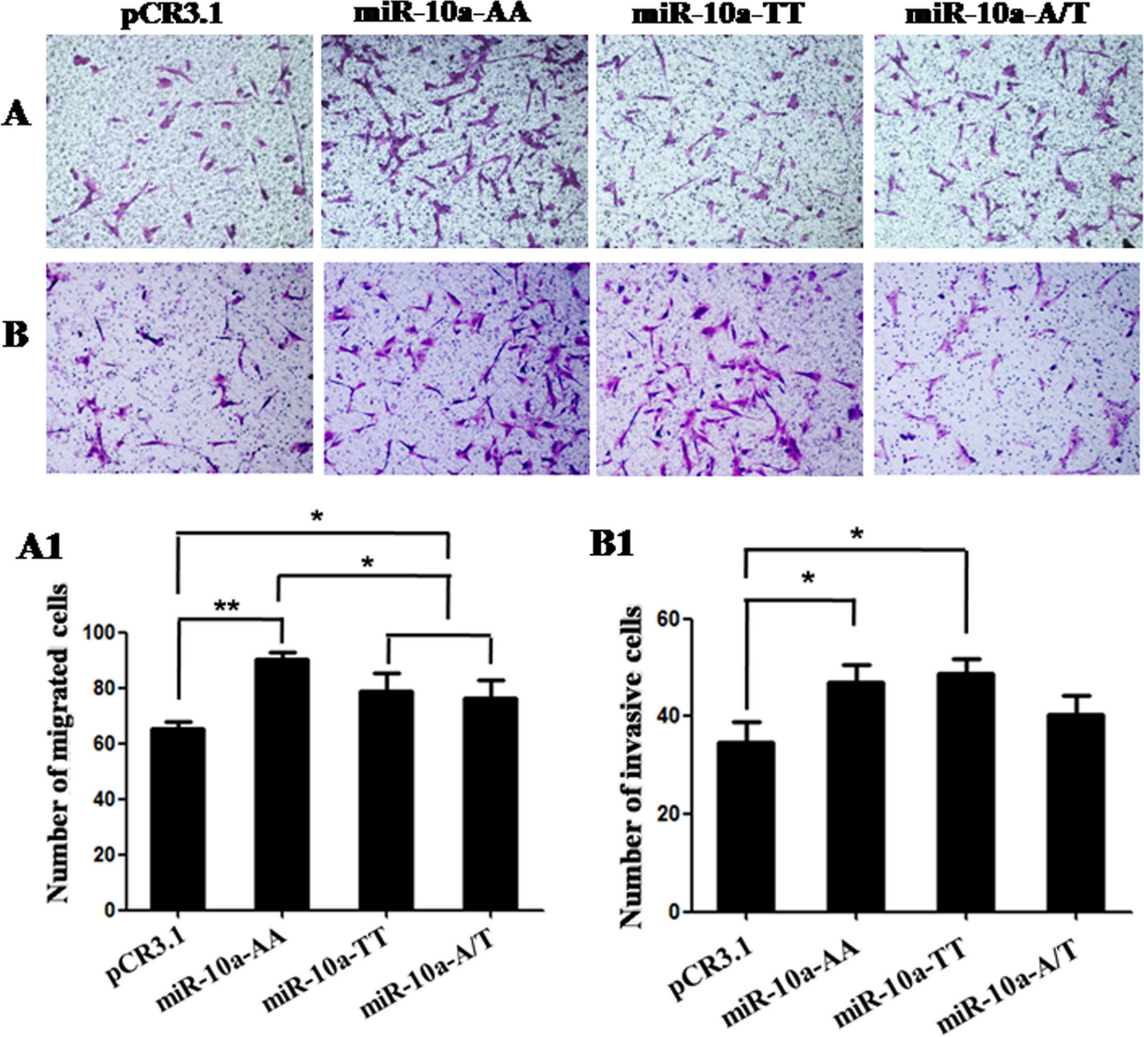
Cell migration and invasion in VCT cells transfected by different genotypes Cells migratory and invasive capacities were detected in VCT cells transfected by pCR3.1, pCR3.1-miR-10a-AA, pCR3.1-miR-10a-TT or pCR3.1-miR-10a-A/T. (**A** and **B**) Photomicrographs of migration and invasion cells 24 h after inoculation. The photographs were shown at x400 original magnification. (**A1** and **B1**) Histogram of number of migration and invasion cells through filters. Data are expressed as the mean numbers of independent triplicate experiments. *P < 0.05, **P < 0.01.

In order to further explore the role of *miR-10a* rs3809783 in controlling cell behavior, we investigated whether the *miR-10a* rs3809783 was associated with the migration and invasion properties of VCT cells. The results showed that AA homozygote significantly promoted cell migratory capacity compared with the empty vector (*P* < 0.01), while TT homozygote and A/T heterozygosity exhibited a significantly lower migration activity than AA homozygote (*P* < 0.05; Figure 4A and 4A1). Similarly, AA homozygote could promote the invasive activity in VCT cells as compared to the empty vector (*P* < 0.05). However, the invasive capacities in TT homozygote and A/T heterozygosity were comparable to AA homozygote (Figure 4B and 4B1).

### *MiR-10a* directly targets the 3′-UTR of *Bim*

**Figure 5:**
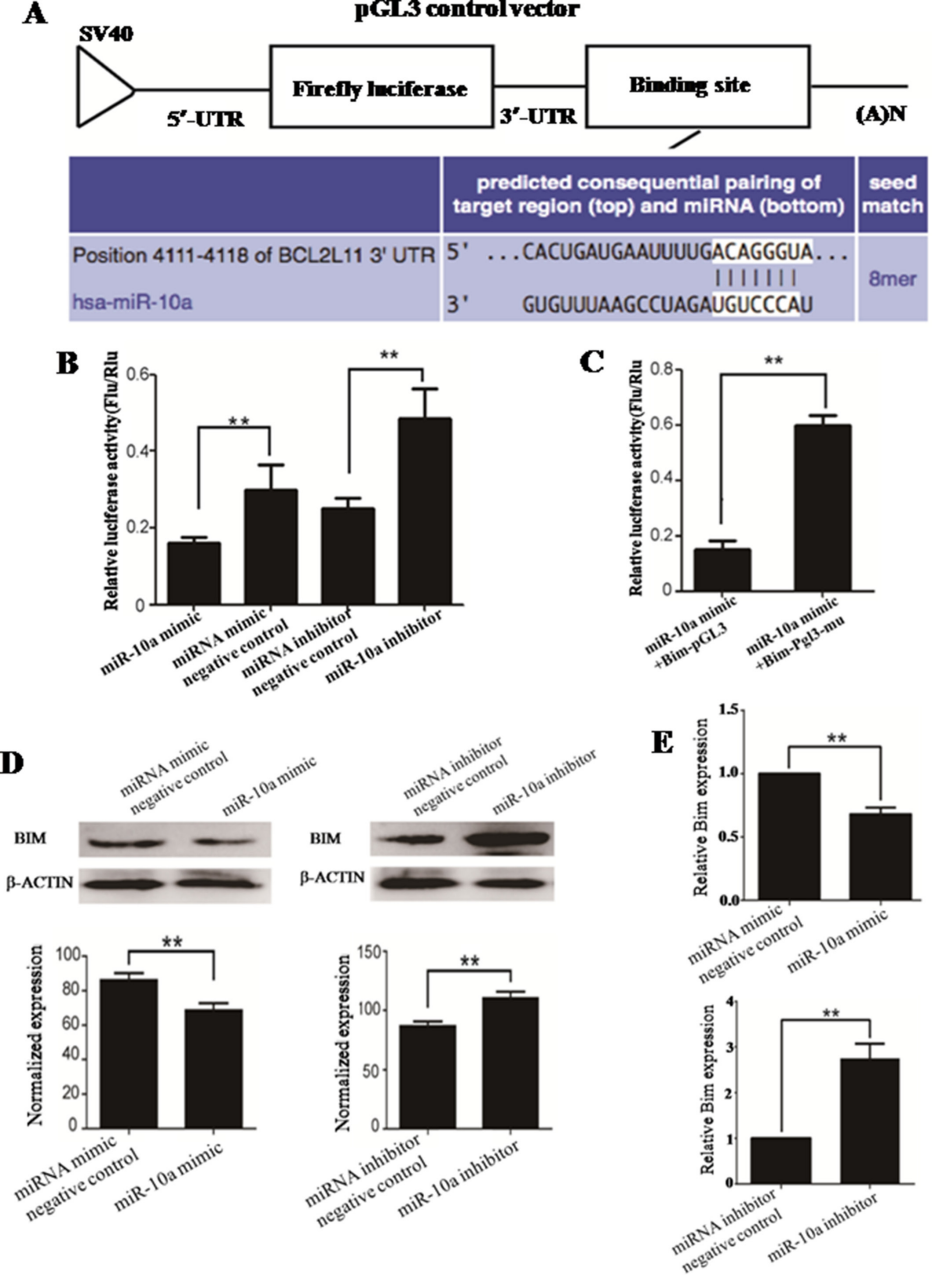
The prediction and confirmation of the *miR-10a* target gene *MiR-10a* binding sites in the 3′-UTR region of *Bim* were compared in cross-species (**A**). HEK-293T cells were co-transfected with *miR-10a* mimic, *miR-10a* inhibitor, miRNA mimic negative control or miRNA inhibitor negative control, and Bim-pGL for dual-luciferase assay (**B**). HEK-293T cells were co-transfected with *miR-10a* and Bim-pGL or Bim-pGL-mu for dual-luciferase assay (**C**). Bim-pGL-mu (mutating putative miR-10a binding region in the 3′-UTR of *Bim*) was used as control. pRL-TK containing Renilla luciferase was co-transfected with 3′-UTR of *Bim* for data normalization. BIM protein level in *miR-10a* mimic or inhibitor-treated cells was detected by western blot (**D**). The bands were analyzed using the Quantity One analyzing system (Bio-Rad). β-ACTIN was served as an internal control. The expression of *Bim* mRNA in *miR-10a* mimic and inhibitor-treated cells were detected by qRT-PCR (E). *Gapdh* was served as an internal reference. **P < 0.01.

The target of *miR-10a* was predicted using target prediction programs online by TargetScan (www.targetscan.org), miRDB (http://mirdb.org/miRDB/index.html) and miRanda (http://www.microrna.org). We chose B-cell lymphoma 2 interacting mediator of cell death (*Bim*) from a large number of putative mRNA targets for the following reasons: *Bim* is a potent pro-apoptotic protein and belongs to the B-cell lymphoma 2 protein family. There is a conservative 7nt *miR-10a* responsive element in 3′-UTR of *Bim* (Figure 5A). Moreover, we found that the expression level of *Bim* was inverted with that of *miR-10a* in HEC-1B, HELA, HEK-293, HEK-293T and VCT cells (Figure 2B).

*MiR-10a* mimic, miRNA mimic negative control, *miR-10a* inhibitor or miRNA inhibitor negative control were separately co-transfected with Bim-pGL (Figure 5B). *MiR-10a mimic is* small, chemically modified double-stranded RNA and designed to mimic endogenous mature *miR-10a* molecule when transfected into cells. MiRNA mimic negative control is a random sequence miRNA mimic molecule that has been validated to not produce identifiable effects on known miRNA function. *MiR-10a* inhibitor is chemically modified, single stranded nucleic acid and designed to specifically bind endogenous mature *miR-10a* and inhibits its expression. MiRNA inhibitor negative control is a random sequence anti-miR molecule that has been validated to produce no identifiable effects on known miRNA function. While cells was transfected with *miR-10a* mimic, the luciferase activity was significantly weakened as compared to miRNA mimic negative control (*P* < 0.01). Compared with miRNA inhibitor negative control, the luciferase activity was obviously strengthened by *miR-10a* inhibitor (*P* < 0.01).

In order to confirm the binding sites of *miR-10a* in the 3′-UTR of *Bim*, the conservative 7nt *miR-10a* responsive element in 3′-UTR of *Bim* was mutated (Figure 5C). The enzyme activity was significantly reduced in cells co-transfected with *miR-10a* mimic and Bim-pGL compared with Bim-pGL-mu (*P* < 0.01), implying that the seed sequence of *miR-10a* could specially recognize *miR-10a* responsive element in the 3′-UTR of *Bim*.

### *MiR-10a* reversely adjusts *Bim* expression *in vitro*

The endogenous *Bim* expression was detected in cells transfected with *miR-10a* mimic or inhibitor by western blot (Figure 5D) and qRT-PCR (Figure 5E). BIM protein level was significantly diminished by *miR-10a* mimic compared with miRNA mimic negative control (*P* < 0.01), while *miR-10a* inhibitor markedly enhanced BIM protein level as compared to miRNA inhibitor negative control (*P* < 0.01). Similarly, *Bim* mRNA level was significantly reduced by *miR-10a* mimic (*P* < 0.01) and raised by *miR-10a* inhibitor (*P* < 0.01).

### Bim is the functional target of *miR-10a*

**Figure 6:**
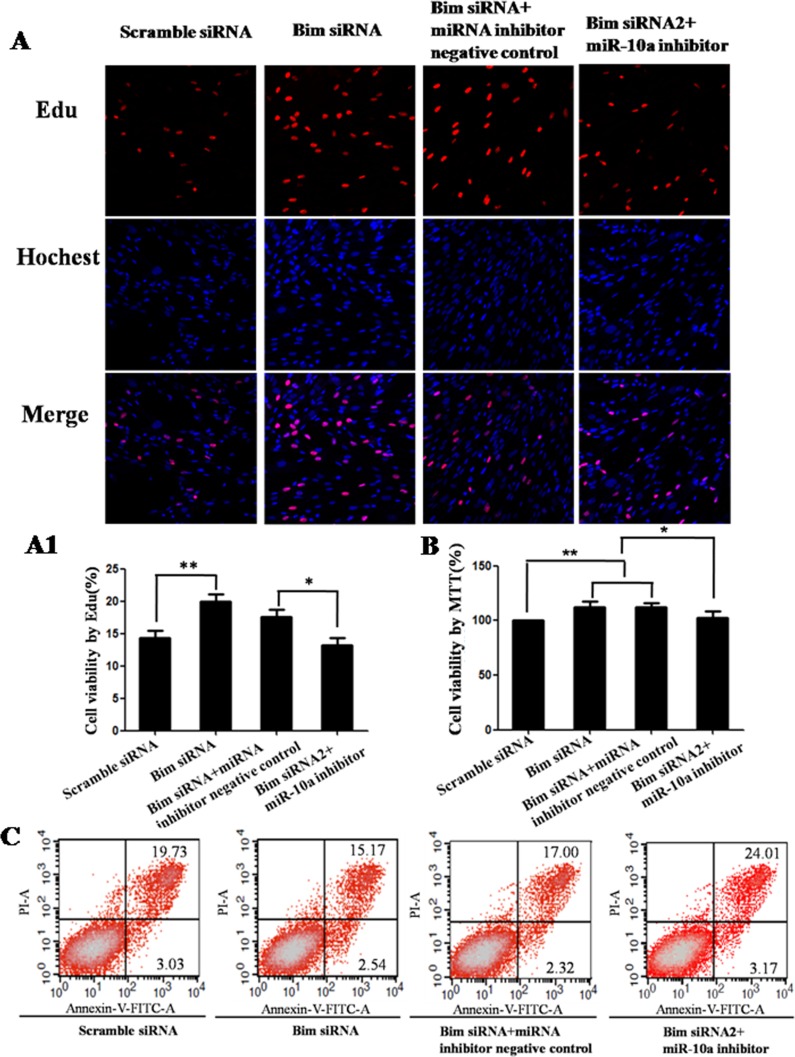
Cell growth analysis in VCT cells transfected by *Bim* siRNA with or without *miR-10a* inhibitor Cell proliferation was determined by EdU assay (**A** and **A1**) and MTT assay (**B**). Red represents the proliferative cells. Blue indicates cell nuclei. The photographs were shown at x400 original magnification. Cell apoptosis was detected by flow cytometry analysis (**C**). Lower left quadrant, viable cells (annexin V-FITC and PI negative); lower right quadrant, early apoptotic cells (annexin V-FITC positive and PI negative); upper right quadrant, late apoptosis/necrosis cells (annexin V-FITC and PI positive). The percentage of early and late apoptotic cells was shown in the lower right and upper right panels, respectively. All experiments were performed at least three times.*P < 0.05, **P < 0.01.

**Figure 7:**
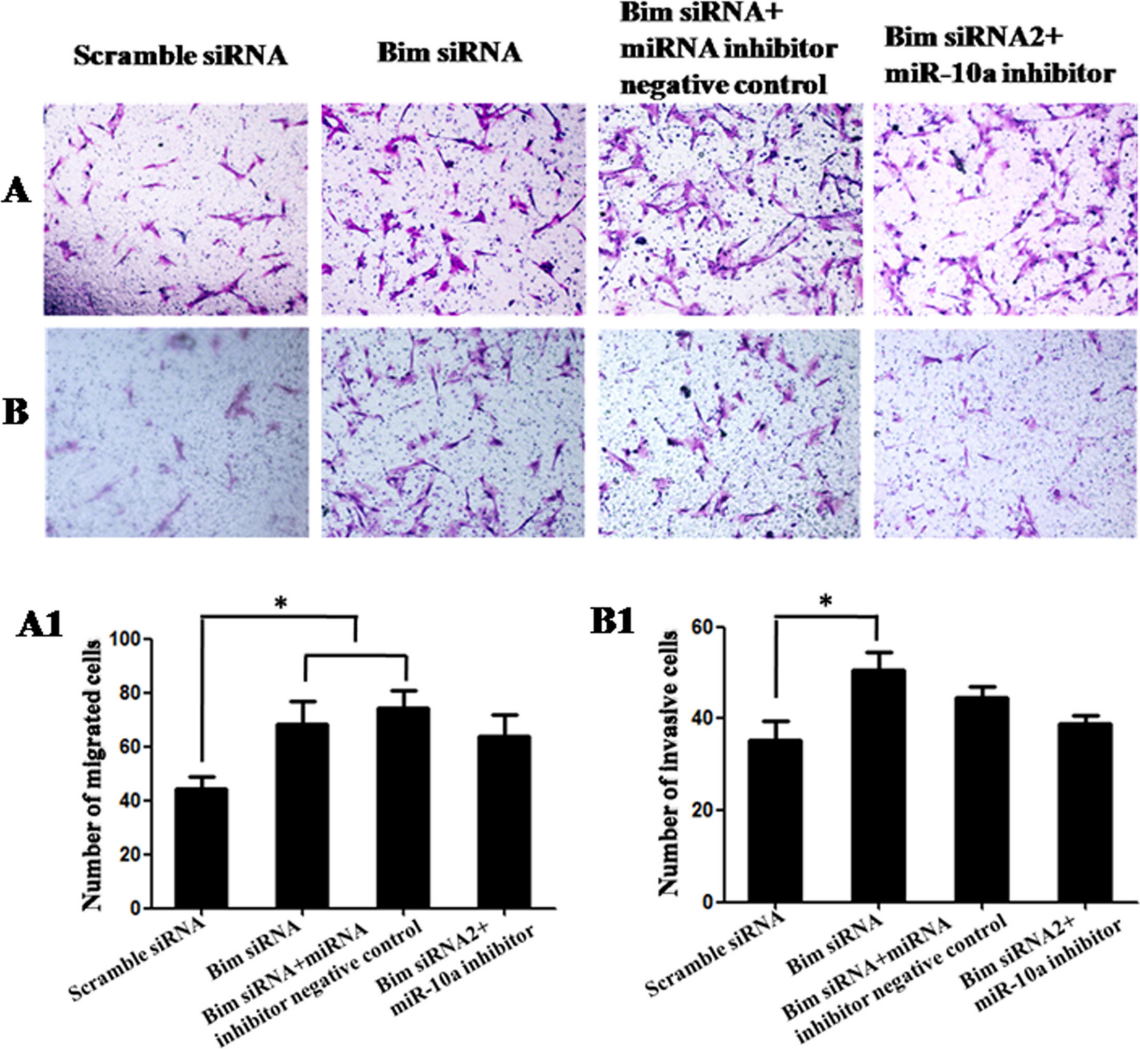
Cell migration and invasion in VCT cells transfected by *Bim* siRNA with or without *miR-10a* inhibitor (**A** and **B**) Photomicrographs of migration and invasion cells 24 h after inoculation. The photographs were shown at x400 original magnification. (**A1** and **B1**) Histogram of number of migration and invasion cells through filters. Data are expressed as the mean numbers of independent triplicate experiments. *P < 0.05.

To determine whether *Bim* was the functional target of *miR-10a*, we first tested the roles of *Bim*. Compared with control, the knockdown of *Bim* promoted cell proliferation detected by Edu (*P* < 0.01; Figure 6A and 6A1) and MTT assay (*P* < 0.05; Figure 6B) and inhibited cell apoptosis detected by flow cytometry analysis (Figure 6C). Also *Bim* knockdown had significant promoting effects on cell migration (*P* < 0.05; Figure 7A and 7A1) and invasion (*P* < 0.05; Figure 7B and 7B1). Then we analyzed whether *miR-10a* low expression could inhibit *Bim* knockdown-mediated the promotion of cell growth and metastasis (Figures 6 and 7). The cell proliferation (*P* < 0.05), migration and invasion capacities were lower and apoptosis level was higher in cells co-transfected with *Bim* siRNA and *miR-10a* inhibitor than that transfected with *Bim* siRNA alone (Figure 9E–9H). Taken together, these results indicate that *miR-10a* executes functions partially by targeting *Bim*.

### A to T substitution in *miR-10a* rs3809783 promotes *Bim* expression

**Figure 8:**
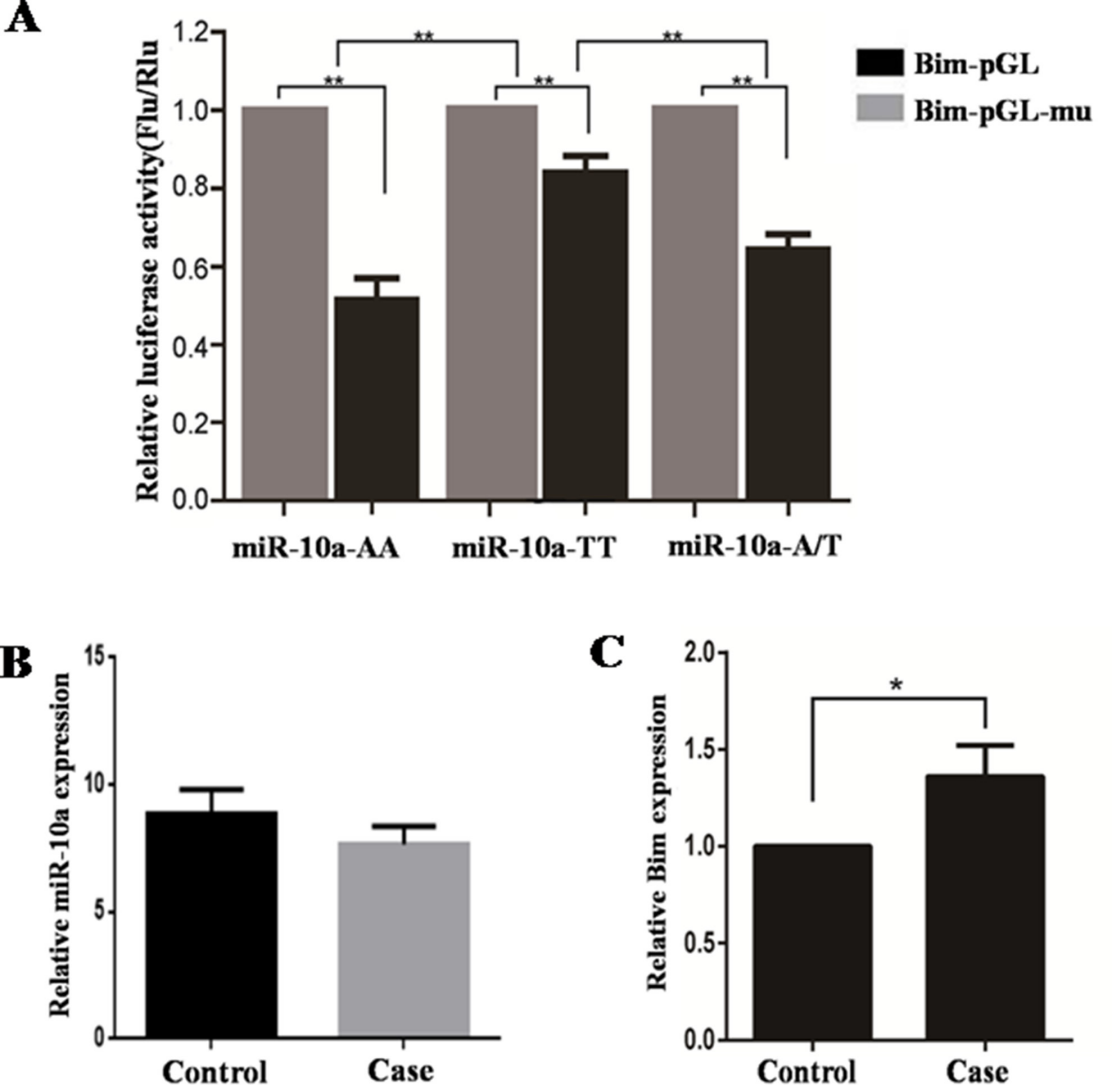
Impact of SNP on *miR-10a* target genes and the expression of *miR-10a* and *Bim* in human decidual tissues The relative luciferase activity of reporter vector with 3′-UTR of *Bim* containing normal or mutated target sites was detected in the presence of pCR3.1-miR-10a-AA, pCR3.1-miR-10a-TT or pCR3.1-miR-10a-A/T (**A**). Bim-pGL-mu was used as control. The expression of *miR-10a* (**B**) and *Bim* (**C**) in the decidual tissues in patients with two or more consecutive missed abortion was detected by qRT-PCR. *U6* and *Gapdh* were served as an internal reference. *P < 0.05, **P < 0.01.

To study whether *miR-10a* rs3809783 affect *Bim* expression, Bim-pGL or Bim-pGL-mu was transfected into cells together with pCR3.1-miR10a-AA, pCR3.1-miR10a-TT or pCR3.1-miR10a-A/T (Figure 8A). Bim-pGL-mu was used as negative control and the different genotypes co-transfected with Bim-pGL-mu showed a higher luciferase activity than with Bim-pGL (*P* < 0.01). When the different genotypes were co-transfected with Bim-pGL, TT homozygote significantly enhanced the luciferase activity compared with AA homozygote (*P* < 0.01) and A/T heterozygosity (*P* < 0.01). The luciferase activity in A/T heterozygosity was similar with that in AA homozygote. All these facts reveal that A to T substitution in *miR-10a* rs3809783 increases *Bim* expression by inhibiting the production of *miR-10a*.

### The dynamics of *miR-10a* and *Bim* expression in the decidual tissues in patients with two or more consecutive missed abortion

The expression levels of *miR-10a* and *Bim* in the decidual tissues from patients with two or more consecutive missed abortion were detected by qRT-PCR (Figure 8B and 8C). Compared with control group, *miR-10a* level was slightly decreased and *Bim* mRNA level was significantly increased (*P* < 0.05) in the decidual tissues in RSA group.

### A to T substitution in the *miR-10a* rs3809783 attenuates the sensibility to progesterone and its antagonist mifepristone

**Figure 9:**
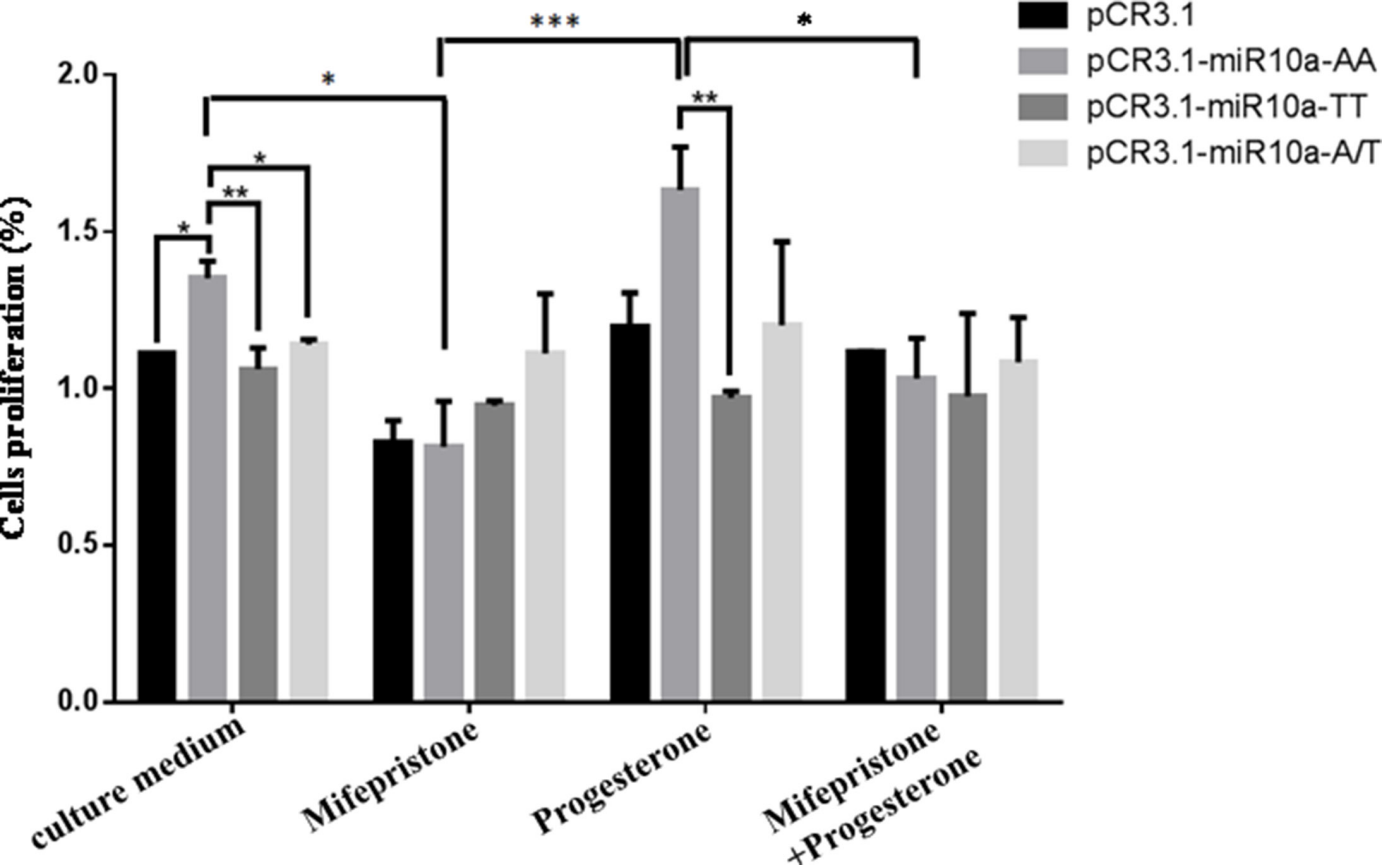
*MiR-10a* rs3809783 attenuates the sensibility to progesterone and mifepristone Cells transfected with pCR3.1-miR-10a-AA, pCR3.1-miR-10a-TT or pCR3.1-miR-10a-A/T were administered with mifepristone or/and pregesterone. The cell proliferation capacity was detected by MTT. *P < 0.05; **P < 0.01; ***P < 0.001.

In order to analyze the sensibility of different genotypes on the abortion and its therapeutic drug, cells transfected with different genotypes were treated by mifepristone or/and pregesterone, and then cell proliferation was detected by MTT assay (Figure 9). The results from MTT assay showed that the AA homozygote promoted the cell viability compared with empty vector (*P* < 0.05) and TT homozygote inhibited cell proliferation as compared to AA homozygote (*P* < 0.01). 50 μM mifepristone treatment inhibited the cell proliferation induced by AA homozygote (*P* < 0.05), but had no significant effect on TT homozygote. AA homozygote-mediated the increase of cell viability was further reinforced by 0.1 μM pregesterone treatment, but had no evident change on TT homozygote. Additionally, pregesterone treatment could partially restore the inhibition of cell proliferation-mediated by mifepristone in cells transfected with AA homozygote, not TT homozygote. All these facts display that A allele is sensitive to mifepristone and progesterone, but T allele is not responsive to mifepristone and progesterone.

## DISCUSSION

In this study, we found the significant association between *miR-10a* rs3809783 and acquiring RSA. Increasing evidences have showed that SNPs in critical components of the miRNA system have a great relationship with some biological functions and diseases [7, 9, 10, 11]. Our survey suggests that the rs3809783 A > T in pri-miR-10a is significantly associated with the increase of the risk of RSA *acquisition* in Han-Chinese population.

The secondary structure prediction of A or T haplotypes showed that A to T substitution in *miR-10a* rs3809783 altered the loop location and descended the predicted ΔG, suggesting the stability of construction was destroyed. Therefore, we speculated that *miR-10a* rs3809783 might diminish the expression level of *miR-10a*. To validate above presumption, the mature *miR-10a* expression level was detected in cells transfected with different genotypes. We found that the expression level of mature *miR-10a* in AA homozygote was markedly higher than that in TT homozygote and A/T heterozygosity, confirming that A to T substitution impeded the production of mature *miR-10a*.

Recently published articles from tumor research have demonstrated that *miR-10a* can effectively modulate cell growth and metastasis [12–14]. In this study, we found that AA homozygote significantly promoted the cell proliferation compared with TT homozygote and A/T heterozygosity. Cell apoptotic level in late stage in TT homozygote were slightly higher than that in AA homozygote. Moreover, AA homozygote had a stronger migration capacity than TT homozygote. All these facts show that normal A allele positively regulate cell proliferation and migration, but rare T allele can attenuate the A allele-mediated reinforcement effects on cell growth and metastasis by inhibiting *miR-10a* expression. The functions of *miR-10a* rs3809783 are similar with the role of *miR-10a* in some tumors. For example, *miR-10a* is overexpressed in pancreatic cancer cells, and the invasiveness of pancreatic cancer cells is decreased by *miR-10a* inhibitor [13]. Taken all together, we speculate that rare T allele may not favorable to endometrial development by inhibiting cell growth, and then result in the increase of the risk of RSA acquisition.

To further explore the possible molecular mechanisms of *miR-10a* rs3809783 executing function, the binding status of *miR-10a* rs3809783 and its target gene was analyzed. *Bim* was confirmed to be the target gene of *miR-10a* by luciferase reporter assay and western blot. Moreover, *Bim* knockdown-mediated the promotion of cell growth was partially attenuated by *miR-10a* low expression. The results further confirmed that *Bim* was the functional target of *miR-10a*. When the 3′-UTR of *Bim* was co-transfected with different genotypes, the luciferase activity was more significantly inhibited by AA homozygote than TT homozygote, suggesting that the A allele could more effectively suppress the expression of *Bim* than T allele. The results were coincidental with our foregoing results of *miR-10a* expression from different genotypes that A allele promoted mature *miR-10a* expression compared with T allele. *Bim* belongs to a pro-apoptotic member of the BCL-2 family and inhibits cell growth. The previous studies have shown that several miRNAs can promote cell proliferation and restrict cell apoptosis by down-regulating *Bim* [15–17]. The homeostasis of apoptosis and proliferation necessarily needs to take place at the maternal endometrium to allow the embryo to invade [18]. The rare T allele in *miR-10a* rs3809783 could diminish cell proliferation ability and enhance cell apoptotic level by up-regulating the expression level of *Bim*. Proliferation defects and excessive cell death are the most likely causes of interrupting endometrial cells reconstruction or endometrium decidualization, which may be able to lead to abnormal embryo implantation or implantation failure [18]. Therefore, we speculate that A to T substitution in *miR-10a* rs3809783 may be able to disrupt the endometrium decidualization, and then result in the occurrence of RSA by up-regulating *Bim*.

In order to further research the possible reasons that A to T substitution in *miR-10a* rs3809783 increased the risk of RSA, we analyzed the sensibility of different genotypes on the abortion and its therapeutic drug. Mifepristone is a progesterone receptor antagonist used as an abortifacient in the first months of pregnancy, which can inhibit cell proliferation [19–21]. Progesterone is beneficial to pregnancy and effective in the treatment of threatened miscarriage due to the decrease of pregesterone [22]. When cells transfected with different genotypes were treated with mifepristone or/and progesterone, AA homozygote-induced cell proliferation was inhibited by mifepristone treatment and further strengthened by progesterone treatment. Also, pregesterone could partially recover the inhibition of cell proliferation-mediated by mifepristone in cells transfected with AA homozygote. However, mifepristone or/and pregesterone treatment did not significantly alter the proliferation capacity in VCT cells transfected with TT homozygote. On basis of these results, we found that A allele was more sensitive to *mifepristone and progesterone* than T allele, *and* A to T substitution attenuated the sensibility of cells to mifepristone and progesterone. Therefore, we speculate that rs3809783 A > T in pri-miR-10a may be able to inhibit the functions of progesterone, and then result in the increase of the risk of RSA acquisition.

In conclusion, this study first discovered that *miR-10a* rs3809783 could increase the risk of RSA *acquisition* in northern Chinese Han population by down-regulating of *miR-10a* level and up-regulating *Bim* expression. These findings may give new insight into understanding of RSA in pathogenesis and mechanism. but also provide an opportunity to resolve the problem of diagnosis and treatment of RSA.

## MATERIALS AND METHODS

### Patients and control samples

Blood samples from 200 patients with two or more consecutive unexplained spontaneous abortions among 13–16weeks were recruited from Peking Union Medical College of China as cases. 200 normal pregnant women (gestational stages were among 13–16 weeks) were recruited from Peking Union Medical College as controls. The decidual tissues from 11 patients with two or more consecutive missed abortion were collected from the Sixth hospital of Shijiazhuang. The decidual tissues from 11 healthy pregnant time-matched women were collected as controls. The healthy pregnant women mainly executed therapeutic abortions because their health or life was threatened by the pregnancy. All recruited persons were Han population and their ancestries lived in North China plain area. Many factors were tested, including virus infection (TORCH) test, T3/T4/TSH, glucose level, immune antibodies (ANA/ACLA) test, and sexual hormones level, the couple's chromosomal karyotype and blood type, the anatomy of reproductive organ and genetic defects or hereditary diseases in their family history. The informed consent was obtained from all participants. The study was approved by Ethics Committee of Research Institute for Family Planning (No. 2011–10).

### Sequencing

DNA was isolated from whole blood of URSA patients and controls, and cells of HEC-1B, HeLa, HEK-293, HEK-293T and villous cytotrophoblast (VCT) using the TIANamp Blood DNA Kit (TIANGEN, Beijing, China). The pri-miR-10a segment in all the DNA specimens was amplified by PCR using the primers as follows: 5′-TGAATCTGACTTCGTGGCAGC-3′ and 5′-CCATAG AGGTGACCCACACAG-3′. The PCR products were sequenced in forward direction with the ABI 3730xl sequencing platform.

### *MiR-10a* expression vectors, cell transfection and treatment

To construct pri-miR-10a expressing vectors, the fragments including pre-miR-10a and its flanking regions were amplified from human genomic DNA using the primers as follows: pri-miR10a-F/HindIII 5′-TTAAGCTTGAATCTGACTT CGTGGC AGC-3′; pri-miR-10a-R/XbaI 5′-CCTCTAGACCATAG AGGTGACCCAC ACAG-3′. The fragments were cloned into the pCR3.1 vector (Invitrogen, Carlsbad, CA, USA) and the constructions were confirmed by direct sequencing. Villous cytotrophoblast (VCT) cells were isolated from villi tissues of pregnant women according to previously described method [23]. All the plasmids were separately transfected into cells with or without 0.1 μM pregesterone or/and 50 μM mifepristone using the lipofectamine 2000 (Invitrogen, Carlsbad, CA, USA) according to the manufacturer's protocol.

### Quantitative reverse-transcriptase polymerase chain reaction (qRT-PCR)

Total RNAs were extracted using Trizol reagent (Invitrogen, Carlsbad, CA, USA). The expression of *miR-10a* was detected by TaqMan miRNA RT-Real Time PCR using TaqMan MicroRNA Reverse Transcription Kit and TaqMan Universal PCR Master Mix (Applied Biosystems, Foster City, CA, USA). *U6* snRNA was used as an endogenous control. The expression of *Bim* was detected by RT-Real-time PCR using iScriptTM cDNA Synthesis Kit (Bio-Rad, Hercules, CA, USA) and FastStart Universal SYBR Green Master (Roche, Mannheim, Germany) with the primers as follows: 5′-AACAAGGAGATGGAACCACTG-3′ (forward) and 5′-CCCGTATAGAGCTGTGAACTC-3′ (reverse). Glyceraldehyde-3-phosphate dehydrogenase (*Gapdh*) was used an endogenous control. Each sample in each group was detected in triplicate. The experiment was repeated three times.

### Edu assay

VCT cells were seeded in a 96-well plate and allowed to attach overnight. The cells were then transfected with pCR3.1-based plasmid, miR-10a-AA, miR-10a-TT, miR-10a-TT/AA, respectively. 48 hours later, cells were carried out by Cell-Light Edu Apollo DNA *in vitro* kit (RiboBio, Guangzhou, China) according to the manufacturer's protocol. Proliferative cells were visualized and imaged under a laser-scanning confocal microscope (CarlZeiss LSM 710 META, Germany). Proliferative cells and total cell number were counted in three fields (magnification ×400) selected in a random manner. The results were described as a ratio of the cell number with proliferative cells *vs* total cells.

### MTT assay

VCT cells were seeded in a 96-well plate and allowed to attach overnight. The cells were then transfected with pCR3.1-based plasmid, miR-10a-AA, miR-10a-TT, miR-10a-TT/AA, respectively. 48 hours later, the cells were incubated with 10 μl MTT (0.5 mg/ml) (Sigma, St. Louis, MO, USA) at 37°C for 4 h. The Formosan was dissolved in dimethyl sulfoxide (Sigma-Aldrich, St. Louis, MO, USA). The absorbance was detected at A_570nm_ with a Model 3550 microplate reader (Bio-Rad, Hercules, CA, USA). Each treatment was repeated thrice within an experiment and the experiment was repeated for three times. The results were described as a ratio of absorbance with different genotypes *vs* empty pCR3.1 control.

### Flow cytometry analysis

VCT cells were harvested after 48 h of transfection. Cell apoptosis was evaluated by annexin V-FITC staining (Inverogen, Carlsbad, CA USA). 5 μl Alexa Fluor^®^ 488 annexin V and 1 μl PI were added into each sample. After 15 min of incubation, 400 μl 1× annexin V-binding buffer was added into ecah sample. The samples were detected at a rate of 8000 events per second by flow cytometry (BD Biosciences, San Jose CA, USA). The experiment was repeated for three times.

### Cell migration and invasion assays

Cell migration and invasion were assayed using a transwell chamber (Corning, NY, USA). VCT cells transfected with pri-miR-10a expressing vectors were placed on the top chamber of 24-well plate with or without 40 μl of 1 mg/ml Matrigel (BD, Franklin Lakes, NJ, USA). After 24 h of incubation, the migrant or invasive cells that had attached to the lower surface of membrane were fixed with 4% formaldehyde and stained with hematoxylin and eosin (Sigma-Aldrich, St. Louis, MO, USA). The cells were imaged using a microscope (BX61; Olympus, Tokyo, Japan). The migration or invasion cells in three different fields (magnification ×400) selected in a random manner were counted and expressed as the average number of cells per field. The experiment was repeated for three times.

### 3′-UTR luciferase reporter assay

The sequence of the 3′-UTR of *Bim* was amplified from human genomic DNA using the primers: Bim-F/KpnI: GGGGTACCCTCTTCTTGGAGATTTTCACTTG and Bim-R/Xhol: AATCTAGAGTCCCTATTTGTCCC TTTGG. The segment was cloned into the downstream of the firefly luciferase gene in pGL3-Control Vector (called Bim-pGL3). Mutating *miR-10a* target sites in the 3′- UTR of *Bim* was used as control (called Bim-pGL3-mu). *MiR-10a* mimic and inhibitor were synthesized by GenePharma (Shanghai, China) and *Bim* siRNA was purchased from RiboBio (Guangzhou, China). The luciferase activity in HEK-293T cells transfected with *miR-10a* mimic or inhibitor and Bim-pGL3 or Bim-pGL3-mu was measured by the Dual-Luciferase Assay (Promega, Madison, WI, USA). All the cells were co-transfected with pRL-TK as data normalization. The experiment was repeated for three times. The results were described as a relative luciferase activity (Firefly LUC/Renilla LUC).

### Western blot

40 μg protein lysates in each sample were boiled in sodium dodecyl sulfate (SDS) sample buffer and then loaded into each lane of 10% polyacrylamide gels. The proteins were transferred to polyvinyldifluoridine (PVDF) membranes (Amersham Pharmacia Biotech, St. Albans, Herts, UK). The membranes were incubated with rabbit anti-BIM polyclonal antibody (Santa Cruz Biotechnology Inc., Santa Cruz, CA USA) and mouse anti-β-ACTIN monoclonal antibody (Santa Cruz Biotechnology Inc., Santa Cruz, CA USA), respectively. The protein signals were detected using the ECL kit (Pierce, Appleton, WI USA). The β-ACTIN was used as an internal control. The bands were analyzed by densitometric evaluation using the Quantity-One software (Bio-Rad, Hercules, CA, USA). The experiment was repeated for three times.

### Statistical analysis

Genotypes and alleles frequencies for the polymorphisms were analyzed using SHESIS software package available from www.shesis.org. All statistical analyses were performed using the SPSS 16.0. All values are expressed as means ± SEM. All histograms were drawn using GraphPad Prism 4.0. Two group's comparison was analyzed by *t*-test. Multiple group's comparisons were analyzed by ANOVA. The level of statistical significance was set at *P* < 0.05.
